# Multivariate sexual selection on male song structure in wild populations of sagebrush crickets, *Cyphoderris strepitans* (Orthoptera: Haglidae)

**DOI:** 10.1002/ece3.736

**Published:** 2013-09-02

**Authors:** Geoffrey D Ower, Kevin A Judge, Sandra Steiger, Kyle J Caron, Rebecca A Smith, John Hunt, Scott K Sakaluk

**Affiliations:** 1 Behavior, Ecology, Evolution and Systematics Section, School of Biological Sciences, Illinois State UniversityNormal, 61790-4120, Illinois; 2 Department of Biological Sciences, Grant MacEwan UniversityEdmonton, Alberta, T5J 4S2, Canada; 3 Institute of Experimental Ecology, University of UlmUlm D-89081, Germany; 4 Centre for Ecology & Conservation, School of Biosciences, University of Exeter in CornwallCornwall, Penryn, TR10 9EZ, U.K

**Keywords:** Communication, fitness surface, mate choice, selection gradient, signal

## Abstract

While a number of studies have measured multivariate sexual selection acting on sexual signals in wild populations, few have confirmed these findings with experimental manipulation. Sagebrush crickets are ideally suited to such investigations because mating imposes an unambiguous phenotypic marker on males arising from nuptial feeding by females. We quantified sexual selection operating on male song by recording songs of virgin and mated males captured from three wild populations. To determine the extent to which selection on male song is influenced by female preference, we conducted a companion study in which we synthesized male songs and broadcast them to females in choice trials. Multivariate selection analysis revealed a saddle-shaped fitness surface, the highest peak of which corresponded to longer train and pulse durations, and longer intertrain intervals. Longer trains and pulses likely promote greater mate attraction, but selection for longer intertrain durations suggests that energetic constraints may necessitate “time outs”. Playback trials confirmed the selection for longer train and pulse durations, and revealed significant stabilizing selection on dominant frequency, suggesting that the female auditory system is tightly tuned to the species-specific call frequency. Collectively, our results revealed a complex pattern of multivariate nonlinear selection characterized primarily by strong stabilizing and disruptive selection on male song traits.

## Introduction

Male acoustic signals play an important role in mate attraction in a number of taxa including birds (Catchpole and Slater 1995), mammals (McComb 1991), anurans (Gerhardt and Brooks 2009), and insects (Alexander 1962; Huber et al. 1989; Robinson and Hall 2002), and are a frequent target of sexual selection (Andersson 1994). Acoustic signaling can impose high energetic costs on males (Prestwich and Walker 1981; Bailey et al. 1993; Hoback and Wagner 1997; Hack 1998), and thus song may also serve as an honest indicator of male quality (Zahavi 1975; Burk 1988). Males that are able to gain access to and efficiently convert resources into attractive sexual advertisements are likely to be of superior quality and achieve a higher mating success. Females may impose directional (linear) sexual selection on males by choosing mates that exhibit the greatest calling effort (e.g., Brooks et al. 2005; Bentsen et al. 2006; Gerhardt and Brooks 2009). Additionally, females may also exert stabilizing (nonlinear) selection through a preference for males that produce song with intermediate structural song characteristics and frequencies (e.g., Brooks et al. 2005; Bentsen et al. 2006). Males that produce song beyond these intermediate thresholds might be expected to have lower reproductive success because they are not recognized as conspecifics or escape detection by the female auditory system.

While extensive research has focused on how sexual selection influences the evolution of acoustic mating signals, up until recently, a majority of experimental studies have investigated only a single song characteristic at a time (e.g., Forrest 1980; Hedrick 1986; Latimer and Sippel 1987; Ritchie 1996; Hunt et al. 2006). This is biologically unrealistic because selection rarely acts individually upon a single trait (Lande and Arnold 1983; Blows 2007). Instead, selection typically acts on combinations of traits that collectively affect an individual's reproductive success (Lande and Arnold 1983). By only investigating single song characteristics in isolation, differences in fitness that are caused by a combination of traits could erroneously be attributed to a single modified trait (Hunt et al. 2009). A better method, yielding a more realistic view of selection, is the use of multiple regression and canonical analysis to measure the direction, shape, and strength of selection acting upon a suite of characters (Lande and Arnold 1983; Phillips and Arnold 1989).

Numerous studies on insects have measured multivariate sexual selection in both the laboratory (e.g., Blows et al. 2004; Brooks et al. 2005; Chenoweth and Blows 2005; Hall et al. 2008; Judge 2010; Gershman et al. 2012) and in wild populations (e.g., LeBas et al. 2003, 2004; Bentsen et al. 2006; Bussière et al. 2008; Punzalan et al. 2010; Robson and Gwynne 2010; Wheeler et al. 2012). While these studies have played an important role in revealing the complexity of sexual selection (Hunt et al. 2009), it is also important to recognize some of the limitations of this approach. First, a limitation common to all conventional selection analyses is that this approach is correlational and therefore any relationship between a trait and fitness may not necessarily be causal. This is especially problematic in field studies where a myriad of external factors are likely to influence selection (Endler 1986) and can be circumvented by experimentally manipulating the sexual traits being examined (e.g., Bentsen et al. 2006; Gerhardt and Brooks 2009). However, despite earlier calls to do so (Endler 1986), relatively few studies have used experimental manipulation to quantify the strength and form of sexual selection (but see Brooks et al. 2005; Bentsen et al. 2006; Gerhardt and Brooks 2009**)**, particularly to confirm the findings of conventional multivariate selection analysis. Second, the biological relevance of selection gradients depend heavily on the estimates of fitness used. In most field studies of sexual selection in insects, pairing success is used as a proxy for fitness (e.g., LeBas et al. 2003, 2004; Bussière et al. 2008; Punzalan et al. 2010; Wheeler et al. 2012) and it is not always known whether males found in copula actually obtain a successful mating (but see Robson and Gwynne 2010). This is almost certainly a consequence of the difficulty in measuring actual male mating success under natural conditions. Sagebrush crickets, *Cyphoderris strepitans* (Orthoptera: Haglidae), offer an excellent model system in this regard because successful mating imposes an unambiguous phenotypic marker on males that results from an unusual form of nuptial feeding by females (Morris 1979; Eggert and Sakaluk 1994).

**Figure 1:**
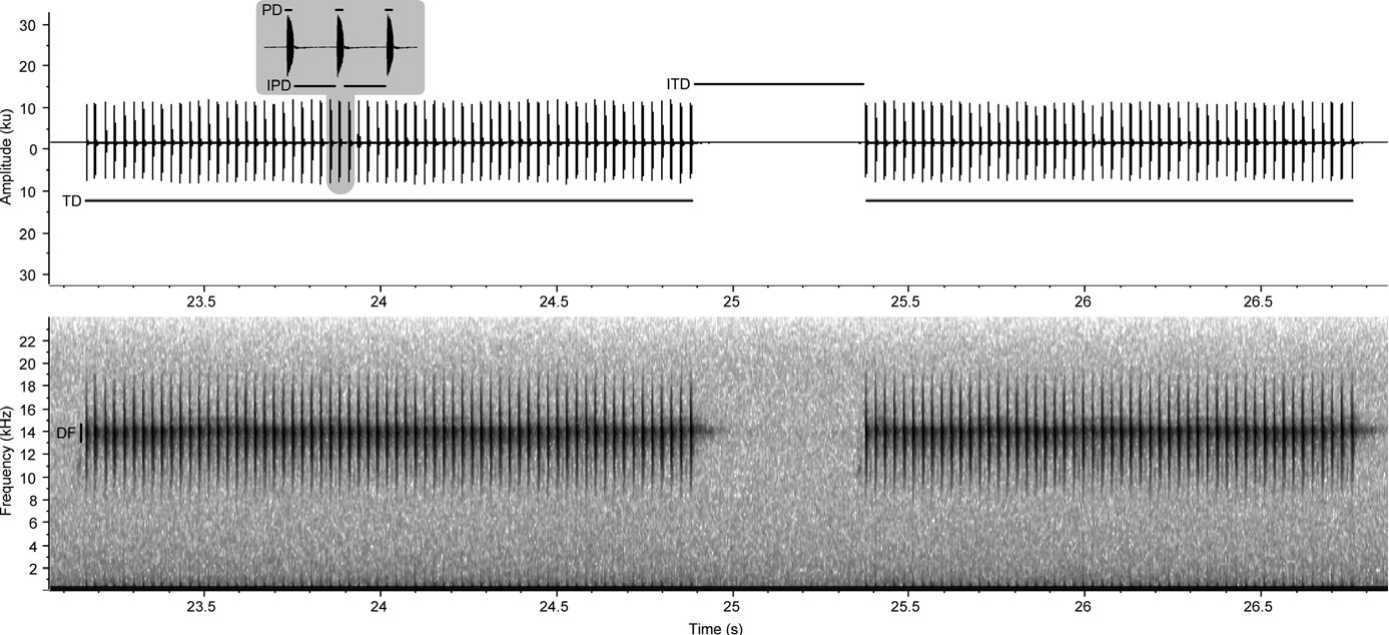
The five focal song characters represent the primary structural characteristics of sagebrush cricket song. Pulse duration (PD) represents a single wing closure and interpulse duration (IPD) is the interval between wing closures. Train duration is the duration of a continuous train of pulses and intertrain duration is the interval between trains. Sagebrush cricket dominant frequency (DF) is approximately 13 kHz as revealed by the spectrogram.

**Figure 2:**
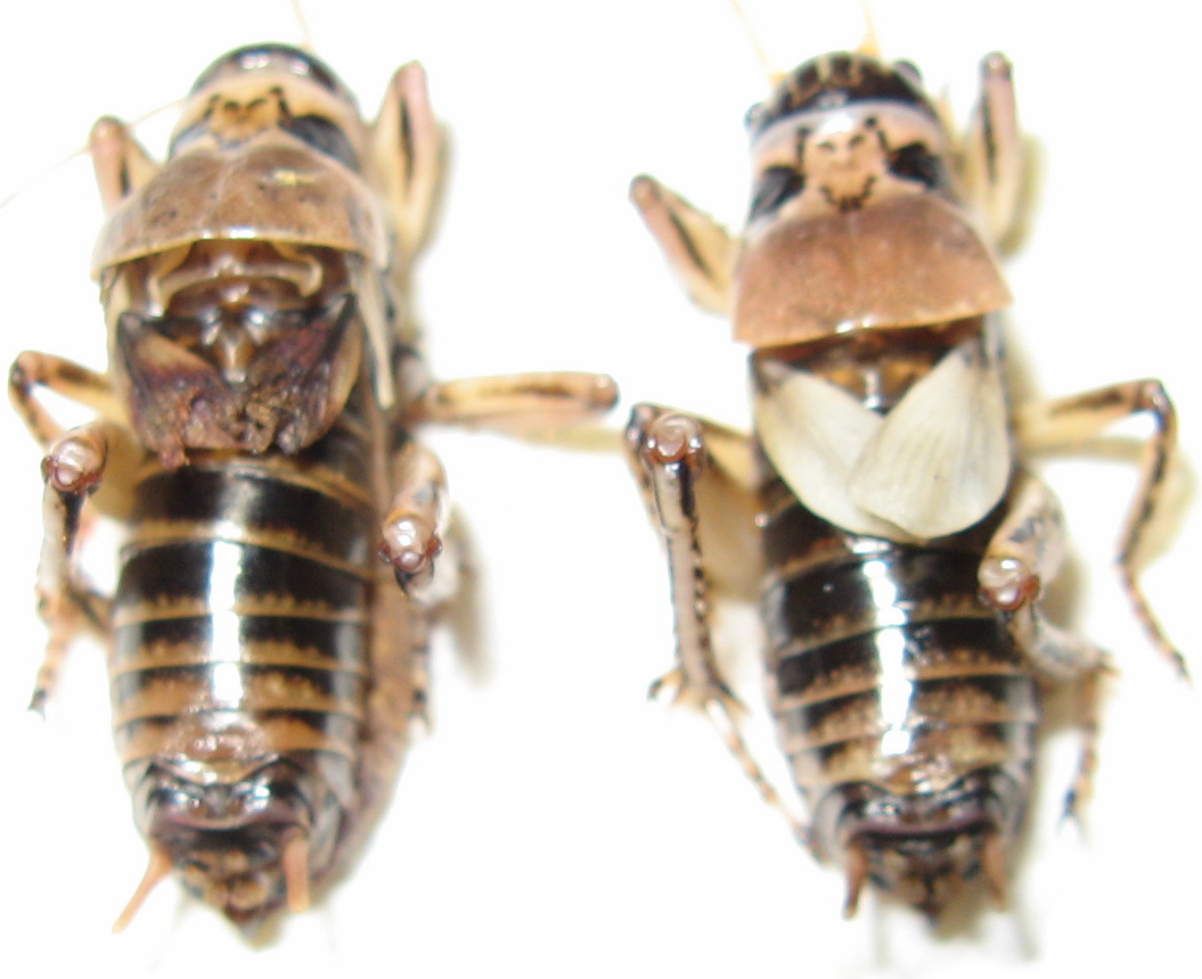
Hind wings of nonvirgin males (left) have melanized wounds resulting from nuptial feeding of females during mating, while virgins have uniformly white, intact hind wings. The forewings, used to produce song, were removed to afford a better view of the hind wings.

The sagebrush cricket, *C. strepitans* (Orthoptera: Haglidae), is one of seven known surviving species of hump-winged grigs, a lineage that was highly diverse between the late Permian to early Cretaceous periods, but is now nearly extinct (Kumala et al. 2005). *C. strepitans* occurs in high-elevation sagebrush meadows nestled within coniferous forests in Wyoming and Colorado (Morris and Gwynne 1978). In Grand Teton National Park where the majority of field studies of *C. strepitans* have been conducted (Sakaluk and Ivy 1999; Sakaluk et al. 2004; Leman et al. 2009), sexual activity commences in mid-May. Each night of the breeding season, males emerge from the ground cover to secure a calling perch in sagebrush or lodgepole pine, from where they sing to attract sexually receptive females (Snedden and Sakaluk 1992; Snedden and Irazuzta 1994). The songs of males have a relatively simple structure comprised of trains of varying lengths made up of pulses emitted from single wing closures (Fig. 1). Once a female has located a male, copulation is initiated when the female climbs onto the dorsum of the male and ends with the transfer of a spermatophore to the female. During copulation, the female feeds on the male's fleshy hind wings and ingests hemolymph seeping from the wounds she inflicts (Morris 1979; Eggert and Sakaluk 1994). The wing-feeding behavior of females provides a powerful tool for documenting male mating success in the wild because the mating status of males can be easily determined by examining their hind wings for the melanized scars resulting from mating (Fig. 2).

An earlier study, in which males were experimentally muted and returned to their natural population, revealed that song is essential to male mating success (Snedden and Sakaluk 1992). However, the features of male song that are subject to selection, and the extent to which male song traits are shaped by sexual acoustic interactions between competing males, female mating preferences, or both, remain unknown. In the congener, *Cyphoderris monstrosa,* territorial males engage in fierce physical contests and employ song in the context of territorial signaling (Mason 1996). In contrast, we have rarely observed physical interactions between male *C. strepitans* (Sakaluk et al. 1995), and although males singing in close proximity occasionally seem to interact acoustically (G. D. O. and S. K. S., pers. obs.), male calling appears to function primarily to facilitate female phonotaxis (Snedden and Irazuzta 1994). In the present study, we attempted to conduct a complete multivariate selection analysis of song traits of males by recording songs of virgin and nonvirgin males captured from three wild populations, and employing multiple regression and canonical analysis to measure the direction, shape, and strength of selection acting upon this suite of sexual characters (Lande and Arnold 1983; Phillips and Arnold 1989). To determine the extent to which sexual selection on male song in the wild is influenced by female song preferences, we conducted a second study in which we synthesized male songs designed to uncouple correlations between male song traits and to capture the full range of phenotypic combinations of song parameters, and broadcast these song types to females in dichotomous choice trials staged in outdoor arenas. By adopting this two-pronged approach, measuring selection on males mating in the wild in concert with experimental manipulation of male song, we aimed to attain a more complete picture of the multivariate sexual selection acting on male sexual signals than hitherto possible using one or the other protocol.

## Methods

Experiments were conducted in Grand Teton National Park, Wyoming, where the high-altitude sagebrush meadow habitat of sagebrush crickets is abundant. Subjects were captured from three populations all found within the Snake River basin: Deadman's Bar (43°45'33.91″N, 110°37'25.12″W), Pacific Creek (43°51'25.67″N, 110°31'8.41″W), and Bridger-Teton (43°54'40.56″N, 110°28'20.24″W). Captured individuals were transported to the University of Wyoming-National Park Service (UW-NPS) Research Station (43°56'23.51″N, 110°38'29.06″W).

### Experiment 1: measuring multivariate sexual selection on male song

#### Sampling populations

We measured sexual selection on the songs of male sagebrush crickets by capturing and recording the songs of virgin and nonvirgin males. Each of the three populations was censused binightly in 2009 by capturing males and inspecting their hind wings for damage to ascertain their mating status (Fig. 2). Once the populations had reached a ratio of approximately 50:50 virgin to mated males, approximately 200 males were captured. This protocol ensured that females were given ample opportunity to mate with the most attractive males in the population.

Captured males were placed into mesh recording chambers and provisioned with a diet of sagebrush galls and sliced apple. On subsequent evenings, the songs of males were digitally recorded directly to a computer for at least 2 min using a unidirectional condenser microphone (BG 4.1; Shure Inc., Niles, IL). On average, males were recorded within 2.7 (±1.1 SD) nights of capture. Raven Pro 1.4 (Cornell Lab of Ornithology, Ithaca, NY) was used to measure the five focal song characters (Fig. 1): pulse duration (PD), interpulse duration (IPD), train duration (TD), intertrain duration (ITD), and dominant frequency (DF).

Because temperature could not be closely controlled during the song recordings (15.7 ± 1.6°C SD), each of the five focal song characters was regressed against temperature, and temperature-adjusted trait values were obtained by adding the residuals to the mean expected trait value. This was necessary because temperature has well-documented effects on sagebrush cricket song and behavior (Morris and Gwynne 1978; Sakaluk and Eggert 2009,) and accounting for the variation attributable to temperature should yield better estimates of the actual selection gradients for the song characters being examined.

#### Multivariate selection analysis

Fitness surfaces were estimated using the multiple regression-based methodology of Lande and Arnold (1983). Mating success of males was scored using a dichotomous fitness measure of 0 for virgins and 1 for nonvirgins. As suggested by Lande and Arnold (1983), the fitness scores were transformed to have a mean of 1 (i.e., fitness was made relative) and the focal song characters were transformed into z-scores with a mean of 0 and a standard deviation of 1. To estimate the standardized linear selection gradients (*β*), a first order multiple regression was fit using the standardized song data as the predictor variables and relative fitness as the response variable. We used a second order quadratic multiple regression model that included all linear, quadratic, and correlational terms to estimate the matrix of standardized nonlinear selection gradients (γ) that describes the curvature of the response surface. Quadratic regression coefficients are known to underestimate by a factor of 0.5 and we therefore doubled the quadratic selection gradients from this model (Stinchcombe et al. 2008).

Interpreting the size and significance of individual γ coefficients can underestimate the strength of nonlinear selection (Phillips and Arnold 1989; Blows and Brooks 2003). Therefore, we examined the extent of nonlinear sexual selection by performing a canonical analysis to locate the major axes (i.e., eigenvectors) of the response surface (Phillips and Arnold 1989). The double regression method of Bisgaard and Ankenman (1996) was used to estimate the strength of linear (**θ**_***i***_) and nonlinear (λ_*i*_) selection along each of the eigenvectors (**m**_**i**_) of the response surface.

As recommended by Mitchell-Olds and Shaw (1987), randomization tests (with 10,000 iterations) were used to assess the significance of all selection gradients, due to nonnormality of the response variable (mating success) and four of the five predictor variables (IPD, TD, ITD, and DF). Randomization tests were also used to assess the significance of linear and nonlinear sexual selection acting on the eigenvectors of γ. For each permutation, the fitness scores were shuffled across cricket calls while the eigenvectors were held constant, which created a null distribution with no relationship between the fitness scores and eigenvectors (see Lewis et al. 2011 for full details of this randomization procedure). Because the eigenvectors were held constant, this procedure differed from that of Reynolds et al. (2010) in which a new canonical rotation is conducted for each iteration (i.e., selection on different eigenvectors are tested at each iteration). We used this approach as we were interested in whether specific eigenvectors experience more linear and nonlinear sexual selection than expected by chance rather than if there was significant linear or nonlinear sexual selection occurring overall (Chenoweth et al. 2012).

#### Fitness surface visualization

Fitness surfaces were visualized by fitting thin-plate splines (Phillips and Arnold 1989; Green and Silverman 1994) to the major axes identified by canonical analysis. Thin-plate splines were estimated using the Tps function of the *fields* package (Furrer et al. 2012) of R (version 2.13.0, http://www.r-project.org/). The smoothing parameter of the Tps function was set to a value that minimized the generalized cross-validation score (Green and Silverman 1994). We plotted the surface in R using both the perspective and contour map views.

#### Comparing the strength and form of sexual selection across populations

We tested whether linear, quadratic, and correlational selection differed among populations using a sequential model building procedure for response surface designs (Draper and John 1988; Chenoweth and Blows 2005). Because the population selection gradients were not significantly different when traits were standardized and fitness made relative within populations (see Results section), the populations were pooled to achieve greater statistical power. In our analysis of pooled data, traits were standardized and relative fitness calculated across populations. Pooling data across populations brought us closer (*n* = 389) to meeting the recommended sample size of 500–1000 desirable for reliably detecting nonlinear selection (Kingsolver et al. 2001).

#### Validating our measurement of sexual selection in the wild

The selection analysis described above assumes that males in the virgin and mated groups had equal opportunities to mate, an assumption that may be violated if males eclose at different times during the breeding season. If mated males are those in the population that eclose earlier and hence have a longer period to obtain matings than males in the virgin group, then differences in the songs of virgin and mated males could simply be an artifact of age-related changes in male morphology as opposed to a causal factor underlying variation in male mating success. Although previous studies of sagebrush crickets emerging within field enclosures erected at Deadman's Bar have suggested that males in the population become sexually active within a few days of each other (Snedden 1996), they cannot rule out the possibility that differences between virgin and mated males are a result of age-related changes (i.e., date of appearance in the population). Accordingly, we conducted a mark-recapture study in 2012 to determine if there is an association between first appearance in the population and the likelihood of mating by males in the field.

At the beginning of the breeding season, we established a study plot at the Bridger-Teton site. This population was chosen because no males had mated at our first population census. On four of the first 5 days of the study, we attempted to capture all of the virgin males calling in the study area and any mated males that had been previously marked. Each male was marked with a numbered tag secured to his pronotum with cyanoacrylic glue. Fluorescent paint was also applied around the pronotum and to the femora to facilitate the recapture of marked individuals with portable fluorescent lanterns. Seven days after the population was first censused, the population attained a ratio of approximately 1:1 virgin to mated males, at which time the study was terminated. We recorded data on the date of initial capture and mating success for 98 males initially marked as virgins.

We used failure-time analysis to examine the effect of a male's capture date on his time to mating (Allison 1995), specifically, a Cox regression as implemented by PROC PHREG in SAS (version 9.2, SAS Institute Inc 2010). The EXACT option was specified in the model statement to handle ties, instances in which different males had the same time to mating. This option was employed because it assumes that mating times are, in reality, continuous and ordered, assumptions that are almost certainly met by our data. The analysis revealed no significant effect of date of capture on time to mating (Wald *χ*^2^ = 0.32, *P* = 0.57). We also compared virgin (*N* = 55) and mated males (*N* = 43) with respect to their date of capture. Mated males were first captured significantly earlier than virgin males (Student's *t*-test with equal variances, *t*_96_ = 2.07, *P* = 0.042), but the difference in the time of their initial appearance in the population was less than half a day (0.49 ± 0.24 days [mean ± SE]). We conclude, therefore, that any differences in the songs of virgin and mated males stems from their effect on male mating success and not from any age-related effects.

### Experiment 2: measuring female preferences for male song

Measuring sexual selection directly on sagebrush cricket song poses a problem, because the very act of mating could alter male song. Mating results in at least a short-term reduction in calling effort due to the refractory interval needed to generate a new spermatophore and to recover from the hemolymph loss associated with nuptial hind wing feeding (Sakaluk et al. 1987, 2004; Sakaluk and Snedden 1990). Hind wing wounding itself does not appear to alter song structure, at least not enough that females are able to detect (Sakaluk and Ivy 1999). However, hind wing wounding may trigger an immune response resulting in an energetically costly immunological trade-off that could cause a reduction in calling effort (Leman et al. 2009). Because even a short-term reduction in singing by nonvirgin males could have influenced the fitness surfaces from Experiment 1, it was necessary to conduct a second experiment in which we directly measured female preference with artificially synthesized songs.

#### Construction of artificial songs

While multivariate selection analysis is designed to handle correlated phenotypic data (Lande and Arnold 1983), strong correlations among traits can still be problematic if it leads to multicollinearity (Mitchell-Olds and Shaw 1987). Multicollinearity can result if only certain combinations of traits are expressed in nature due to an evolutionary history of persistent selection that removes unfit phenotypes from the population (Brooks et al. 2005). Estimating selection gradients based on these restricted subsets of naturally occurring characters could result in biased estimates of selection gradients or even prevent them from being detected (Mitchell-Olds and Shaw 1987; Brooks et al. 2005), because females may prefer acoustic signals that are biophysically impossible for males to produce (Ryan and Rand 2003). To address these problems, we synthesized artificial signals that were designed to be phenotypically uncorrelated (e.g., Brooks et al. 2005; Bentsen et al. 2006).

Using a procedure outlined in Brooks et al. (2005), 100 artificial songs (henceforth referred to as manipulated songs) were created that were specially designed so that the five focal song characters (Fig. 1) maintained their measured univariate means and standard deviations from Experiment 1 (Table 1), but were uncorrelated with one another. For each focal song character, random normal distributions with a mean of 0 and standard deviation of 1 were independently drawn using the rnorm function in the R statistical package (R Development Core Team 2012). These randomized *z*-scores represent the number of standard deviations an observation would be from the mean song character. To transform the *z*-scores back to the original song character distributions, the *z*-scores were multiplied by the measured song character standard deviation and added to the measured song character mean (Table 1). A composite control song was created by setting each of the five focal song characters to their measured mean values from Experiment 1 (Table 1).

**Table 1:** Means and standard deviations of song traits of males captured in the wild

	Recorded songs		Manipulated songs		Composite control
		
	Mean	SD	Mean	SD	
Pulse duration (msec)	3.268	0.480	3.261	0.478	3.266
Interpulse duration (msec)	27.698	2.495	26.760	2.694	27.089
Train duration (msec)	929.833	745.923	1366.763	808.311	950.992
Intertrain duration (msec)	5020.211	3749.538	6740.460	3723.345	5222.860
Dominant frequency (KHz)	13.055	0.782	12.994	0.794	13.049

Manipulated song trait means and standard deviations for synthesized songs broadcast to females in choice trials approximated their respective recorded means and standard deviations of wild males, but were generated from independently drawn, randomized univariate normal distributions. The composite control was set approximately equal to mean trait values of wild males.

Songs were synthesized using Adobe Audition 3. To avoid any potential effect of female temporal or spatial bias in the arenas, the manipulated and control songs were assigned random start times (between 0.0 sec and 1.0 sec), and randomly assigned to the left or right stereo channel of the generated WAV sound files. The synthesized songs were transferred to MiniMee Media Players (MEElectronics Inc., Walnut, CA) that were used to play the songs in the arenas.

#### Female phonotaxis trials

Females were captured from Deadman's Bar in 2010 and brought back to the UW-NPS research station where they were placed into cages and fed a diet of Purina Cat Chow, bee pollen, and sliced apple. This higher quality diet allowed the females to be kept in captivity long enough to conduct the trials without their condition deteriorating.

Arena choice trials were conducted at a field site near the UW-NPS research station, where there are no naturally occurring sagebrush crickets, making it a suitable location for conducting phonotaxis trials without bioacoustical interference from nonexperimental individuals. Four arenas were built separated by 30.5 m, which was a sufficient distance for the dense vegetation of the field site to acoustically isolate the arenas from one another. The arenas were built by hammering rebar into the ground and burying stainless steel roof flashing around the perimeter of a 2.0 m × 0.3 m area. Custom built plywood speaker towers were placed at the terminal ends of the arena, which positioned the stereo speakers (VS2620; Altec Lansing Inc., New York, NY) at the average sagebrush height of 0.52 m (Snedden 1996) and within the natural height range at which males sing (Sakaluk and Eggert 2009). The speaker towers also sheltered the electronics from rain and snow during the trials. Because females of many orthopteran species have been shown to prefer louder males (Bailey et al. 1990), we ensured that each speaker was broadcasting at 100 dB measured with a digital sound level meter (33-2055; Radioshack Inc., Forth Worth, TX) held 5 cm away, the approximate amplitude of natural sagebrush cricket song (Morris and Gwynne 1978).

To minimize behavioral disturbance, holes were molded into the ground of the same dimensions as the cages in which females were housed. This ensured that once a cage was placed in the hole, the soil inside the cage was flush with the soil in the arena so that females could climb out of the cages of their own volition without being disturbed (Snedden and Irazuzta 1994). At the start of a trial, the cage lids were carefully loosened so that they could gently be removed after the female had been given 5 min to acclimate to the manipulated and control signals being broadcast from the speakers. Females were observed throughout the trials to ensure that they were displaying phonotaxis behavior. If a female approached within 0.3 m of a speaker that was broadcasting a manipulated signal, it was scored 1 for attractive (or 0 for unattractive if she chose the control) and the trial was ended. If a female did not choose a signal within 30 min, the trial was ended and repeated on the subsequent evening until the female made a choice.

### Statistical analysis

Selection analyses were conducted using the same methods as in Experiment 1. However, due to inclement weather conditions throughout the entire field season in 2010, we were unable to capture a sufficient number of females (*n* = 25 in total) to test the 100 synthesized signals independently. This necessitated using each female in four trials, which created a problem of statistical nonindependence. To test whether individual female choice biased the selection gradients, we used a bootstrap resampling procedure following Brooks et al. (2005) in which we randomly drew one independent observation from each of the 25 females, and calculated the selection gradients based on this sample. This process was repeated 10,000 times, which built independent distributions for each selection gradient that could be compared with the observed selection gradients from pooled trials. If any of the observed gradients were biased by individual song preferences of the females, we would expect them to fall within the tails of their respective distribution of independent gradients. We calculated *P* values by summing the number of |independent gradients| that were greater than their respective |observed gradient| and dividing by 10,000. None of the *P* values were significant (*P* = 0.514–0.998), so individual trials involving the same female were treated as independent in further analyses.

We compared the strength and form of sexual selection acting on songs of males captured in the field and synthesized songs broadcast to females in choice trials using the same sequential model building procedure used to evaluate differences between populations (Draper and John 1988; Chenoweth and Blows 2005). This procedure allowed the strength of standardized selection gradients between studies to be statistically tested, but it cannot be used to compare fitness surfaces across studies as canonical analysis places each study in its own unique canonical space. Therefore, to compare differences in sexual selection across the two studies in canonical space we calculated the angle between the dominant vectors of selection in both studies (**m**_**1**_ for the field study and **m**_**5**_ for our manipulative study) following the protocol outlined in Lewis et al. (2011). We calculated the 95% credible interval for this angle using a Bayesian approach (R code provided on request to GDO) implemented in the *MCMCglmm* package of R (version 2.13.0) (Hadfield 2010).

## Results

### Experiment 1: measuring sexual selection on male song

Partial *F* tests from sequential modeling showed no significant difference in linear (*F*_5,377_ = 0.371, *P* = 0.869), quadratic (*F*_5,367_ = 0.128, *P* = 0.986), or correlational (*F*_10,347_ = 1.21, *P* = 0.283) selection gradients among our three study populations, allowing the populations to be pooled for greater statistical power.

Standardized linear, quadratic, and correlational selection gradients are presented in Table 2A. There was no significant linear selection operating on any song trait. There was, however, quadratic selection operating on DF and significant positive correlational selection between PD and IPD, and between TD and ITD (Table 2A). No other correlational selection gradients were significantly different from zero.

**Table 2:** The vector of standardized linear selection gradients (*β*) and the matrix of standardized quadratic and correlational selection gradients for the five focal songs characters of (A) songs of males captured in the wild and (B) synthesized songs broadcast to females in choice trials

β		γ				

		PD	IPD	TD	ITD	DF
A. Songs of males captured in the wild						
PD	0.092 ± 0.048	0.092 ± 0.037				
IPD	0.025 ± 0.049	0.101 ± 0.051*	0.013 ± 0.026			
TD	−0.013 ± 0.051	0.024 ± 0.067	−0.126 ± 0.084	0.031 ± 0.023		
ITD	−0.010 ± 0.052	0.117 ± 0.066	0.067 ± 0.055	0.302 ± 0.134*	0.105 ± 0.039	
DF	0.064 ± 0.048	−0.033 ± 0.042	0.010 ± 0.045	0.034 ± 0.082	−0.020 ± 0.063	−0.076 ± 0.027*
B. Synthesized songs broadcast to females						
PD	−0.017 ± 0.106	0.233 ± 0.084				
IPD	0.091 ± 0.106	−0.005 ± 0.119	−0.136 ± 0.083			
TD	0.159 ± 0.107	0.078 ± 0.137	0.057 ± 0.112	0.104 ± 0.096		
ITD	−0.076 ± 0.106	0.067 ± 0.115	−0.153 ± 0.110	−0.100 ± 0.141	0.093 ± 0.088	
DF	0.084 ± 0.107	0.106 ± 0.168	−0.182 ± 0.130	0.257 ± 0.111*	−0.141 ± 0.106	−0.359 ± 0.074*

PD, pulse duration; IPD, interpulse duration; TD, train duration; ITD, intertrain duration; DF, dominant frequency.

Randomization tests: *P < 0.05, **P < 0.01, ***P < 0.001.

**Figure 3:**
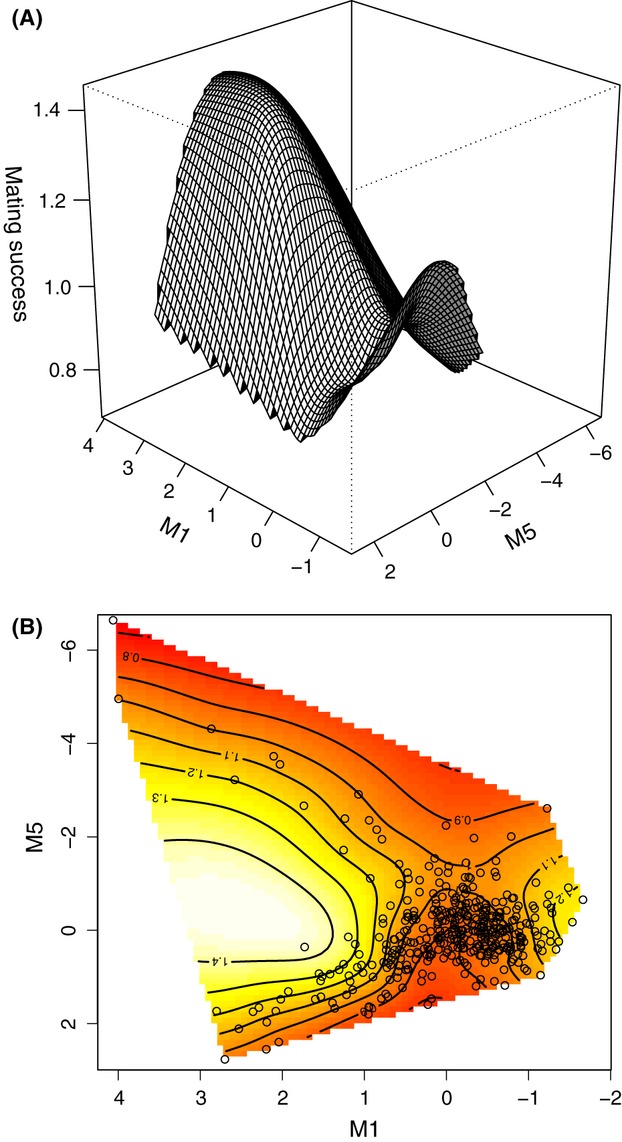
Thin-plate spline (A) perspective view and (B) contour map visualization of the two major axes of nonlinear selection (**m**_**1**_ and **m**_**5**_) operating on songs of males captured in the wild. Each point on the contour plot represents an individual male.

**Figure 4:**
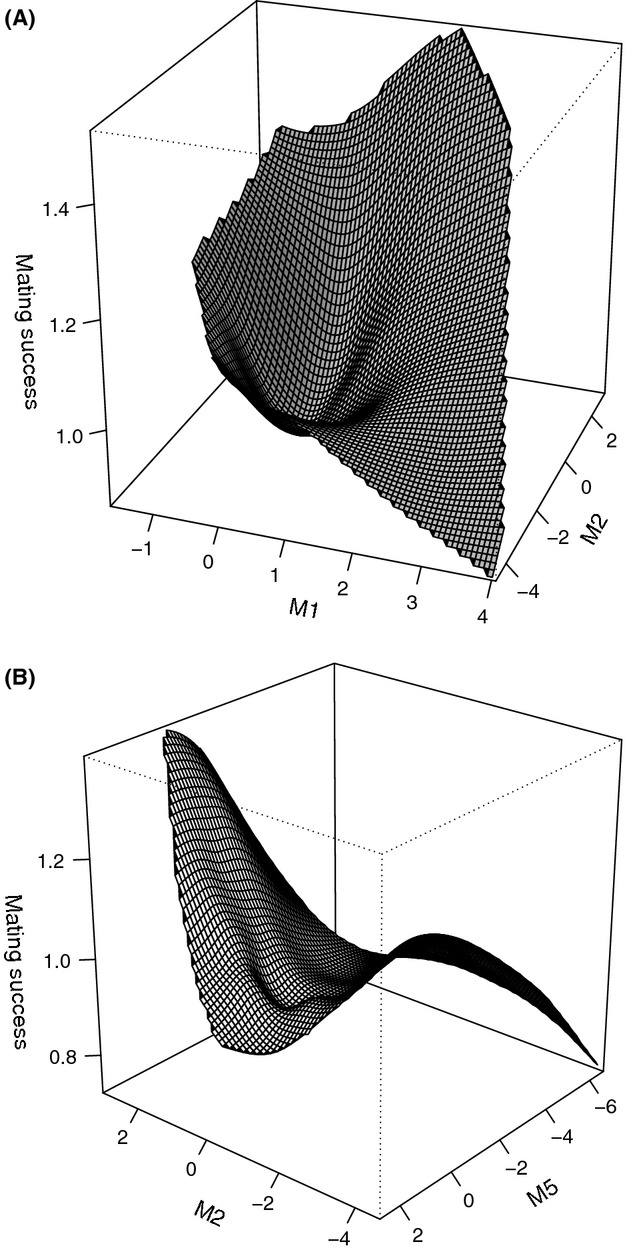
Thin-plate spline visualizations (perspective view only) of nonlinear selection operating on songs of males captured in the wild. (A) **m**_**1**_ and **m**_**2**_ (B) **m**_**2**_ and **m**_**5**_.

Canonical rotation of the γ matrix resulted in three eigenvectors with significant nonlinear sexual selection, two with positive eigenvalues (**m**_**1**_ and **m**_**2**_) and one with a negative eigenvalue (**m**_**5**_) (Table 3A). The dominant eigenvector of disruptive selection (**m**_**1**_) was heavily influenced by negative weightings from TD and ITD, and to a lesser extent, a negative weighting from PD (Table 3A). The dominant eigenvector of stabilizing selection (**m**_**5**_) was heavily loaded by a negative weighting from TD and a positive weighting from ITD, and to a lesser extent, a negative weighting from IPD (Table 3A). The complex, saddle-shaped nature of nonlinear sexual selection along these two dominant eigenvectors can be visualized in Figure 3. The second eigenvector of disruptive selection (**m**_**2**_) was positively weighted by PD and IPD and, to a lesser degree, negatively weighted by TD (Table 3A); sexual selection along this eigenvector can be visualized in Figure 4. There was no significant linear sexual selection (**θ**_**i**_) along any of the major eigenvectors of the fitness surface identified by canonical analysis (Table 3A).

**Table 3:** Linear (θ_*i*_) and nonlinear (λ_*i*_) selection gradients and the M matrix of eigenvectors from the canonical analysis of γ for (A) songs of males captured in the wild and (B) synthesized songs broadcast to females in choice trials

θ_*i*_		λ_*i*_	*M*				

			PD	IPD	TD	ITD	DF
A. Songs of recorded males							
**m_1_**	−0.014 ± 0.056	0.203 ± 0.060***	−0.323	−0.014	−0.601	−0.730	0.010
**m_2_**	0.073 ± 0.050	0.095 ± 0.039*	0.642	0.650	−0.392	0.025	−0.105
**m_3_**	−0.002 ± 0.044	−0.020 ± 0.024	−0.538	0.606	−0.005	0.238	0.535
**m_4_**	0.089 ± 0.046	−0.043 ± 0.023	0.440	−0.230	0.101	−0.263	0.821
**m_5_**	0.002 ± 0.051	−0.153 ± 0.042***	−0.014	−0.397	−0.689	0.583	0.168
B. Arena female choice trials							
**m_1_**	0.159 ± 0.100	0.181 ± 0.075*	0.539	0.038	0.674	−0.335	0.375
**m_2_**	−0.120 ± 0.108	0.134 ± 0.070	0.693	−0.265	−0.205	0.637	−0.033
**m_3_**	0.020 ± 0.104	0.011 ± 0.081	−0.461	−0.566	0.451	0.426	0.288
**m_4_**	0.069 ± 0.106	−0.060 ± 0.076	−0.088	0.649	0.464	0.497	−0.329
**m_5_**	0.046 ± 0.114	−0.299 ± 0.079***	−0.093	0.433	−0.291	0.230	0.817

Randomization tests: *P < 0.05, **P < 0.01, ***P < 0.001.

### Experiment 2: measuring female preferences for male song

Female choice trials revealed no significant linear selection on the five male song characters (Table 2B). There was significant negative quadratic selection on DF indicating stabilizing selection, but the other standardized quadratic selection gradients were not significantly different from zero (Table 2B). There was significant positive correlational selection between TD and DF, but none of the other standardized correlational gradients were significant (Table 2B).

**Figure 5:**
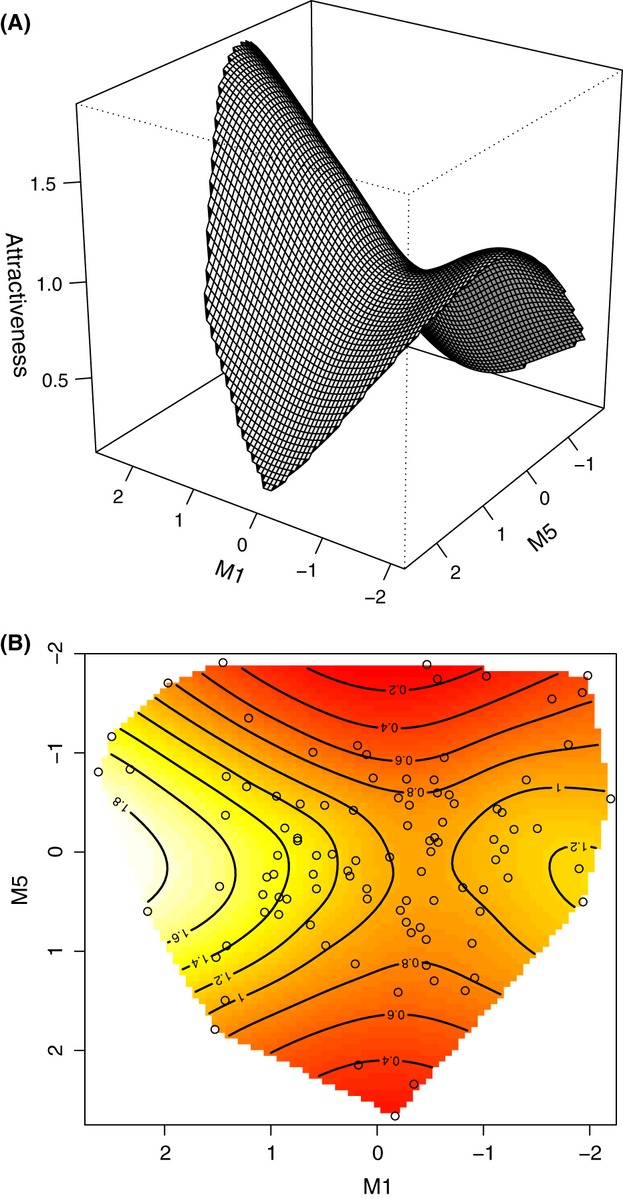
Thin-plate spline (A) perspective view and (B) contour map visualization of the two major axes of nonlinear selection (**m**_**1**_ and **m**_**5**_) operating on synthesized songs broadcast to females in choice trials. Each point on the contour plot represents a synthetic song.

Canonical analysis of γ identified two eigenvectors with significant nonlinear sexual selection, **m**_**1**_ and **m**_**5**_ (Table 3B). The dominant eigenvector of nonlinear sexual selection (**m**_**5**_) was characterized by a negative eigenvalue, indicative of stabilizing selection acting along this eigenvector, and was most heavily weighted by DF (Table 3B). The remaining eigenvector (**m**_**1**_) had a positive eigenvalue, indicative of disruptive sexual selection operating along this eigenvector, with the strongest weightings coming from positive contributions of PD and TD (Table 3B). This mixture of a positive and negative eigenvalues demonstrates that the fitness surface is best described as a multivariate saddle and this can be visualized using thin-plate splines in Figure 5. There was no significant linear sexual selection (**θ**_**i**_) along any of the major eigenvectors of the fitness surface identified by canonical analysis (Table 3B).

Partial *F* tests from sequential modeling showed no significant difference in linear (*F*_5,477_ = 0.764, *P* = 0.576), quadratic (*F*_5,467_ = 0.974, *P* = 0.433), or correlational (*F*_10,447_ = 1.474, *P* = 0.146) selection gradients for songs of males captured in the field and synthesized songs broadcast to females in choice trials. The angle between the dominant eigenvectors of nonlinear sexual selection (**m**_**1**_ for the field study and **m**_**5**_ for our manipulative study) was 89.94° (95% credible intervals: 69.87°–109.60°) suggesting that these dominant eigenvectors are almost orthogonal in canonical space. Consequently, although our sequential model building approach showed little difference in the standardized selection gradients for call parameters across experiments, the large angle between the dominant eigenvectors suggests that the multivariate combination of call parameters under the strongest sexual selection differs significantly across our experiments.

## Discussion

Our field study revealed significant nonlinear sexual selection on song traits of male sagebrush crickets captured from wild populations. Specifically, there was significant disruptive selection acting on **m**_**1**_ and significant stabilizing selection on **m**_**5**_, the two major axes of nonlinear selection, resulting in the saddle-shaped selection surface depicted in Figure 3. The major feature of this rotated fitness surface is a rising ridge that peaks at high values of **m**_**1**_, corresponding to longer TDs and PDs, and longer intertrain intervals. Longer trains and pulses may be favored because the increased sound output they represent leads to increased mate attraction, but the fact that longer ITDs are also favored suggests an energetic constraint. Males capable of producing sustained call trains may have to take longer “time outs” to recover from these bouts of intense energy expenditure. Such a trade-off is also evidenced by the positive correlational selection acting on TD and ITD. This interpretation is further bolstered by the results of the playback experiment, the analysis of which also revealed significant nonlinear selection on male song. Just as in the field results, disruptive selection on one of the major axes of nonlinear selection (**m**_**1**_) and stabilizing selection on another (**m**_**5**_) produced a saddle-shaped fitness surface with a rising ridge peaking at high levels of **m**_**1**_ (Fig. 5). High values of **m**_**1**_ in playback trials also correspond to longer train and PDs, but in contrast to field results, shorter ITDs. In effect, high values of durations **m**_**1**_ represent greater sound output and calling effort and, consistent with previous studies, such songs may be favored because they result in greater passive attraction of females, are actively preferred by females, or both (Holzer et al. 2003; Brooks et al. 2005; Bentsen et al. 2006). However, songs used in the playback experiment were deliberately constructed so that different song traits were randomly selected to capture the entire range of phenotypic combinations of song parameters, even those that may be biologically implausible. Thus, although songs with longer train (and pulse) durations and shorter intertrain intervals may confer the highest fitness, there may be few, if any males capable of optimizing both song parameters owing to energetic constraints or biomechanical limitations imposed by their forewings. Under these circumstances, the best male strategy may be to produce longer TDs at the cost of having to take slightly longer respites from calling.

In addition to **m**_**1**_, a second minor axis of disruptive selection emerged from our field study, **m**_**2**_, high values of which correspond to long PDs and increased IPDs, and to a lesser extent, shorter TDs. Inspection of the fitness surfaces in Figure 4 suggests that maximal fitness is attained at high levels of **m**_**2**_. Thus, longer PDs appear to be favored at the expense of increased time between pulses. Longer PD (and chirp rate) has been shown to be associated with increased oxygen consumption in the variable field cricket, *Gryllus lineaticeps* (Hoback and Wagner 1997). If, in fact, longer train and PDs are more energetically costly, this would be consistent with the theory that energetically costly signals are attractive to females because they are honest indicators of male quality (Zahavi 1975; Burk 1988; Welch et al. 1998). Indeed, the saddle-shaped fitness surfaces obtained in both the field and playback studies appears consistent with signaling theory (Getty 1998), which posits that attraction of females at a greater than linear rate per unit investment in the signal enforces the honesty of the signal. An alternative explanation is that the ordinary least square regression-based approach of Lande and Arnold (1983) predisposes the fitness surface to take this saddle-shaped form (Shaw and Geyer 2010). More work is needed, however, to test between these alternatives (Hunt and Sakaluk in press).

Examination of the standardized gradients from the field study suggests that dominant carrier frequency is subject to stabilizing sexual selection (Table 2B). This finding was reinforced by canonical analysis of γ from playback trials. Specifically, there was significant stabilizing selection acting on **m**_**5**_, the most dominant eigenvector of nonlinear sexual selection, which was most heavily weighted by DF. This suggests that the auditory system of females is most sensitive to the DF of male song, 13.0 kHz (±0.6 kHz). The auditory system of *C. strepitans* has not been characterized, but hearing in the congener *C. monstrosa* is based on a primitive two channel hearing system with auditory receptors of two types: low-tuned receptors most sensitive to 2 kHz signals and broadly tuned receptors that respond to a full range of frequencies up to (and possibly above) 20 kHz, including the 12 kHz DF of *C. monstrosa* song (Mason et al. 1999). Differential activation of these two auditory receptor types allows *C. monstrosa* to crudely discriminate between high and low frequency signals, but they are apparently incapable of discerning fine frequency differences between conspecific signals (Mason et al. 1999). In contrast to *C. monstrosa*, selection for intermediate dominant frequencies resulting from female phonotactic preferences suggests that *C. strepitans* has a more fine-tuned auditory system that is capable of perceiving frequency differences among signals of competing males. The quadratic selection gradient for DF was −0.359 (Table 2B), which is more than three times stronger than the typical range of values between −0.1 and 0.1 reported in many previous studies (Kingsolver et al. 2001).

Although our sequential model building approach showed little difference in the standardized selection gradients for call parameters across the field study and the playback experiment, the large angle between the dominant eigenvectors suggests that the multivariate combination of call parameters under the strongest sexual selection in the field study differs significantly from that of the playback study. Selection documented in the playback study can only be attributed to female song preferences because in playback trials, synthetic signals were used, thus completely eliminating any influence of male–male signaling interactions. In contrast, the pattern of selection revealed in the field study could, in theory, be attributed to signaling interactions between males occurring in the context of territorial disputes, female song preferences, or both. As noted earlier, acoustic interactions between neighboring males do occur in *C. strepitans*, but we do not know which songs traits are important in resolving acoustic contests. In house crickets, temporal aspects of males' aggressive songs are correlated with their ability to win fights (Brown et al. 2006). Similarly, certain features of male sagebrush cricket song may be important in mediating territorial disputes, but if that were the case, song traits favored in the context of male–male competition might differ from those favored by female mating preferences. Thus, the difference in the pattern of multivariate sexual selection revealed in the two studies may be due to selection imposed through male–male signaling interactions in the field. Our study also demonstrates the utility of combining measurements of selection in unmanipulated wild populations with studies in which the male trait of interest is experimentally manipulated. Although playback trials revealed that females prefer song traits closely aligned with greater sound output and calling effort, the pattern of nonlinear selection documented for field-collected males suggests that males in nature face energetic constraints that physically preclude them from producing the phenotypically most attractive trait combinations. Thus, studies focusing solely on the manipulation of single song parameters in isolation may result in misleading inferences concerning the form and strength of selection acting collectively on traits.

It is worth noting that neither the field study, nor experimental playback trials, detected any significant directional selection acting on any of the song traits, in contrast to previous studies of field crickets (Holzer et al. 2003; Brooks et al. 2005; Bentsen et al. 2006), but similar to a study by Hall et al. (2008) examining postcopulatory sexual selection in the Australian field cricket *Teleogryllus commodus*. Thus, had our study focused on strictly on linear selection, we would have concluded erroneously that sexual selection does not act on male song structure. While many studies have measured selection acting on sexual signals in insects, it has only been more recently that studies have conducted a complete multivariate selection analysis, including canonical rotation, to examine the strength and form of sexual selection that female mating preferences impose on males (review in Hunt and Sakaluk in press). Those that have invariably have revealed a complex pattern of selection on male sexual traits that, with one exception (Brooks et al. 2005), have produced fitness surfaces characterized by the multivariate saddle of the kind documented in the present study (Blows et al. 2003; Bentsen et al. 2006; Hall et al. 2008; Judge 2010; Gershman et al. 2012; Hunt and Sakaluk in press).

No difference was found in linear, nonlinear, or correlational selection gradients among the three study populations. The populations are located relatively close together along the Snake River and its tributary, Pacific Creek, with only 7.4 km separating the Bridger-Teton and Pacific Creek populations, and 13.6 km between Pacific Creek and Deadman's Bar. Because our populations are relatively close together and share very similar habitats, they likely are exposed to similar predation pressure and environmental conditions, which could explain the similar fitness surfaces in these populations. There also may be undiscovered backcountry sagebrush cricket populations that could facilitate sufficient gene flow between populations to offset any differences. Alternatively, our study populations could be relicts of a much larger population that historically inhabited the broader Grant Teton region and have not had sufficient time to diverge. The lack of differences in sexual selection gradients among populations further indicates that female preferences for male song likely will not be a driving factor in the future divergence of these populations, unless female preferences change within populations.

In conclusion, our study, which involved measures of male mating success in wild populations along with experimental manipulation of male song played back to females in dichotomous mate choice trials, revealed a complex pattern of multivariate nonlinear selection characterized primarily by strong stabilizing and disruptive selection on male song traits; correlational selection was weak or nonexistent. There was also no evidence of significant directional selection on any song characters. Our study cautions against the use of strictly manipulative studies of male sexual signals to measure selection resulting from female choice because while such studies can reveal the trait combinations females prefer, selection on male traits in wild populations will ultimately reflect physical and energetic constraints that may preclude certain phenotypic variants. However, the use of such studies in concert with measurement of selection on sexual signals of males in natural populations can help disentangle the relative contributions of male competition and female mate choice to the evolution of signal structure. Like Bentsen et al. (2006), we also caution against laboratory studies that draw inferences about sexual selection based on the manipulation of sexual signals in a single dimension, as these will invariably underestimate the strength and complexity of sexual selection acting on male sexual traits.

## References

[b1] Alexander RD (1962). Evolutionary change in cricket acoustical communication. Evolution.

[b2] Allison D (1995). Survival analysis using the SAS system: a practical guide.

[b3] Andersson M (1994). Sexual selection.

[b4] Bailey WJ, Cunningham RJ, Lebel L (1990). Song power, spectral distribution and female phonotaxis in the bushcricket, *Requena verticalis* (Tettigoniidae: Orthoptera): active female choice or passive attraction. Anim. Behav.

[b5] Bailey WJ, Withers PC, Endersby M, Gaull K (1993). The energetic costs of calling in the bushcricket *Requena verticalis* (Orthoptera: Tettigoniidae: Listroscelidinae). J. Exp. Biol.

[b6] Bentsen CL, Hunt J, Jennions MD, Brooks R (2006). Complex multivariate sexual selection on male signaling in a wild population of *Teleogryllus commodus*. Am. Nat.

[b7] Bisgaard S, Ankenman B (1996). Standard errors for the eigenvalues in second-order response surface models. Technometrics.

[b8] Blows MW (2007). A tale of two matrices: multivariate approaches in evolutionary biology. J. Evol. Biol.

[b9] Blows MW, Brooks R (2003). Measuring nonlinear selection. Am. Nat.

[b10] Blows MW, Brooks R, Kraft PG (2003). Exploring complex fitness surfaces: multiple ornamentation and polymorphism in male guppies. Evolution.

[b11] Blows MW, Chenoweth SF, Hine E (2004). Orientation of the genetic variance-covariance matrix and the fitness surface for multiple male sexually selected traits. Am. Nat.

[b12] Brooks R, Hunt J, Blows MW, Smith MJ, Bussière LF, Jennions MD (2005). Experimental evidence for multivariate stabilizing sexual selection. Evolution.

[b13] Brown WD, Smith AT, Moskalik B, Gabriel J (2006). Aggressive contests in house crickets: size, motivation and the information content of aggressive songs. Anim. Behav.

[b14] Burk T (1988). Acoustic signals, arms races and the costs of honest signaling. Fla. Entomol.

[b15] Bussière LF, Gwynne DT, Brooks R (2008). Contrasting sexual selection on males and females in a role-reversed swarming dance fly, *Rhamphomyia longicauda* Loew (Diptera: Empididae). J. Evol. Biol.

[b16] Catchpole CK, Slater PJB (1995). Bird song: biological themes and variations.

[b17] Chenoweth SF, Blows MW (2005). Contrasting mutual sexual selection on homologous signal traits in *Drosophila serrata*. Am. Nat.

[b18] Chenoweth SF, Hunt J, Rundle HD, Svensson EI, Calsbeek R (2012). Analyzing and comparing the geometry of individual fitness surfaces. The adaptive landscape in evolutionary biology.

[b19] Draper NR, John JA (1988). Response-surface designs for quantitative and qualitative variables. Technometrics.

[b20] Eggert A-K, Sakaluk SK (1994). Sexual cannibalism and its relation to male mating success in sagebrush crickets, *Cyphoderris strepitans* (Orthoptera: Haglidae). Anim. Behav.

[b21] Endler JA (1986). Natural selection in the wild.

[b22] Forrest TG (1980). Phonotaxis in mole crickets: its reproductive significance. Fla. Entomol.

[b23] Furrer R, Nychka D, Sain S (2012). Fields: tools for spatial data. Version 6.6.3.

[b24] Gerhardt CH, Brooks R (2009). Experimental analysis of multivariate female choice in gray treefrogs (*Hyla versicolor*): evidence for directional and stabilizing selection. Evolution.

[b25] Gershman SN, Mitchell C, Sakaluk SK, Hunt J (2012). Biting off more than you can chew: sexual selection on the free amino acid composition of the spermatophylax in decorated crickets. Proc. R. Soc. B.

[b26] Getty T (1998). Handicap signaling: when fecundity and viability do not add up. Anim. Behav.

[b27] Green PJ, Silverman BW (1994). Nonparametric regression and generalised linear models.

[b28] Hack MA (1998). The energetics of male mating strategies in field crickets (Orthoptera: Gryllinae: Gryllidae). J. Insect Behav.

[b29] Hadfield JD (2010). MCMC methods for multi-response generalized linear mixed models: the MCMCglmm R package. J. Stat. Software.

[b30] Hall MD, Bussière LF, Hunt J, Brooks R (2008). Experimental evidence that sexual conflict influences the opportunity, form and intensity of sexual selection. Evolution.

[b31] Hedrick AV (1986). Female preferences for male calling bout duration in a field cricket. Behav. Ecol. Sociobiol.

[b32] Hoback WW, Wagner WE (1997). The energetic cost of calling in the variable field cricket, *Gryllus lineaticeps*. Physiol. Entomol.

[b33] Holzer B, Jacot A, Brinkhof MWG (2003). Condition-dependent signaling affects male sexual attractiveness in field crickets, *Gryllus campestris*. Behav. Ecol.

[b34] Huber F, Moore TE, Loher W (1989). Cricket behavior and neurobiology.

[b35] Hunt J, Sakaluk SK, Shuker DM, Simmons LW Mate choice. The evolution of insect mating systems.

[b36] Hunt J, Jennions MD, Spyrou N, Brooks R (2006). Artificial selection on male longevity influences age-dependent reproductive effort in the black field cricket *Teleogryllus commodus*. Am. Nat.

[b37] Hunt J, Breuker CJ, Sadowski JA, Moore AJ (2009). Male-male competition, female mate choice and their interaction: determining total sexual selection. J. Evol. Biol.

[b38] Judge KA (2010). Female social experience affects the shape of sexual selection on males. Evol. Ecol. Res.

[b39] Kingsolver JG, Hoekstra HE, Hoekstra JM, Berrigan D, Vignieri SN, Hill CE (2001). The strength of phenotypic selection in natural populations. Am. Nat.

[b40] Kumala M, McLennan DA, Brooks DR, Mason AC (2005). Phylogenetic relationships within hump-winged grigs, *Cyphoderris* (Insecta, Orthoptera, Tettigonioidea, Haglidae). Can. J. Zool.

[b41] Lande R, Arnold SJ (1983). The measurement of selection on correlated characters. Evolution.

[b42] Latimer W, Sippel M (1987). Acoustic cues for female choice and male competition in *Tettigonia cantans*. Anim. Behav.

[b43] LeBas NR, Hockham LR, Ritchie MG (2003). Nonlinear and correlational sexual selection on “honest” female ornamentation. Proc. R. Soc. B.

[b44] LeBas NR, Hockham LR, Ritchie MG (2004). Sexual selection in the gift giving dance fly, *Rhamphomyia sulcata*, favors small males carrying small gifts. Evolution.

[b45] Leman JC, Weddle CB, Gershman SN, Kerr AM, Ower GD, St John JM (2009). Lovesick: immunological costs of mating to male sagebrush crickets. J. Evol. Biol.

[b46] Lewis Z, Wedell N, Hunt J (2011). Evidence for strong intralocus sexual conflict in the Indian meal moth, *Plodia interpunctella*. Evolution.

[b47] Mason AC (1996). Territoriality and the function of song in the primitive acoustic insect *Cyphoderris monstrosa* (Orthoptera: Haglidae). Anim. Behav.

[b48] Mason AC, Morris GK, Hoy RR (1999). Peripheral frequency mis-match in the primitive ensiferan *Cyphoderris monstrosa* (Orthoptera: Haglidae). J. Comp. Physiol. A.

[b49] McComb KE (1991). Female choice for high roaring rates in red deer, *Cervus elaphus*. Anim. Behav.

[b50] Mitchell-Olds T, Shaw RG (1987). Regression analysis of natural selection: statistical inference and biological interpretation. Evolution.

[b51] Morris GK (1979). Mating systems, paternal investment and aggressive behavior of acoustic Orthoptera. Fla. Entomol.

[b52] Morris GK, Gwynne DT (1978). Geographical distribution and biological observations of *Cyphoderris* (Orthoptera: Haglidae) with a description of a new species. Psyche.

[b53] Phillips PC, Arnold SJ (1989). Visualizing multivariate selection. Evolution.

[b54] Prestwich KN, Walker TJ (1981). Energetics of singing in crickets: effect of temperature in three trilling species (Orthoptera: Gryllidae). J. Comp. Physiol. B.

[b55] Punzalan D, Rodd FH, Rowe L (2010). Temporal variation in patterns of multivariate sexual selection in a wild insect population. Am. Nat.

[b56] R Development Core Team (2012). R: a language and environment for statistical computing.

[b57] Reynolds RJ, Childers DK, Pajewski NM (2010). The distribution and hypothesis testing of eigenvalues from the canonical analysis of the gamma matrix of quadratic and correlational selection gradients. Evolution.

[b58] Ritchie MG (1996). The shape of female mating preferences. Proc. Natl Acad. Sci. USA.

[b59] Robinson DJ, Hall MJ (2002). Sound signaling in Orthoptera. Adv. Insect Physiol.

[b60] Robson LJ, Gwynne DT (2010). Measuring sexual selection on females in sex-role-reversed Mormon crickets (*Anabras simplex*, Orthoptera: Tettigoniidae). J. Evol. Biol.

[b62] Ryan MJ, Rand AS (2003). Sexual selection in female perceptual space: how female túngara frogs perceive and respond to complex population variation in acoustic mating signals. Evolution.

[b63] Sakaluk SK, Eggert A-K (2009). Coping with the cold: temperature and mating activity of male sagebrush crickets *Cyphoderris strepitans* (Orthoptera: Haglidae). Physiol. Entomol.

[b64] Sakaluk SK, Ivy TM (1999). Virgin-male mating advantage in sagebrush crickets: differential male competitiveness or non-independent female mate choice?. Behaviour.

[b65] Sakaluk SK, Snedden WA (1990). Nightly calling durations of male sagebrush crickets, *Cyphoderris strepitans*: size, mating and seasonal effects. Oikos.

[b66] Sakaluk SK, Morris GK, Snedden WA (1987). Mating and its effect on acoustic signaling behavior in a primitive orthopteran, *Cyphoderris strepitans* (Haglidae): the cost of feeding females. Behav. Ecol. Sociobiol.

[b67] Sakaluk SK, Snedden WA, Jacobson KA, Eggert A-K (1995). Sexual competition in sagebrush crickets: must males hear calling rivals?. Behav. Ecol.

[b68] Sakaluk SK, Campbell MTH, Clark AP, Johnson JC, Keorpes PA (2004). Hemolymph loss during nuptial feeding constrains male mating success in sagebrush crickets. Behav. Ecol.

[b69] SAS Institute Inc (2010). SAS/STAT® 9.2 user's guide.

[b70] Shaw RG, Geyer CJ (2010). Inferring fitness landscapes. Evolution.

[b71] Snedden WA (1996). Lifetime mating success in male sagebrush crickets: sexual selection constrained by a virgin male mating advantage. Anim. Behav.

[b72] Snedden WA, Irazuzta S (1994). Attraction of female sagebrush crickets to male song: the importance of field bioassays. J. Insect Behav.

[b73] Snedden WA, Sakaluk SK (1992). Acoustic signaling and its relation to male mating success in sagebrush crickets. Anim. Behav.

[b74] Stinchcombe JR, Agrawal AF, Hohenlohe PA, Arnold SJ, Blows MW (2008). Estimating nonlinear selection gradients using quadratic regression coefficients: double or nothing?. Evolution.

[b75] Welch AM, Semlitsch RD, Gerhardt HC (1998). Call duration as an indicator of genetic quality in male gray tree frogs. Science.

[b76] Wheeler J, Gwynne DT, Bussière LF (2012). Stabilizing sexual selection for female ornaments in a dance fly. J. Evol. Biol.

[b77] Zahavi A (1975). Mate selection—a selection for a handicap. J. Theor. Biol.

